# Isolation of Exosomes from MDA-MB-231 Cells Using a Paddle Screw System and Detection of TNBC-Associated Exosomal miRNAs

**DOI:** 10.3390/mi17030362

**Published:** 2026-03-16

**Authors:** Han Sol Kim, Soo Suk Lee

**Affiliations:** Department of Pharmaceutical Engineering, Soonchunhyang University, 22 Soonchunhyang-ro, Shinchang-myeon, Asan-si 31538, Chungcheongnam-do, Republic of Korea; itig2000@sch.ac.kr

**Keywords:** exosome, microRNA, MDA-MB-231 cell line, 3D-paddle screw, Bi-directional extension (BDE), RT-qPCR

## Abstract

Exosomes are nanoscale extracellular vesicles that carry disease-associated microRNAs (miRNAs) and represent promising biomarkers for cancer diagnosis. Triple-negative breast cancer (TNBC) lacks well-defined molecular markers, necessitating sensitive and integrable analytical approaches for TNBC-related exosomal miRNAs. In this study, exosomes were isolated from MDA-MB-231 TNBC cells using a paddle screw-based system designed to enhance mass transfer through active rotation, providing a mechanically driven isolation strategy that is compatible with miniaturized and microfluidic platforms. This dynamic isolation process enabled rapid and efficient exosome recovery within a short processing time. Three TNBC-associated miRNAs encapsulated in the isolated exosomes were quantitatively analyzed using polyadenylation tailing (poly(A) tailing) and specific bidirectional extension sequence-based assays combined with reverse transcription quantitative real-time PCR (RT-qPCR). The bidirectional extension (BDE) assay generated highly specific PCR templates, leading to improved amplification specificity and reduced background signals. The RT-qPCR analysis exhibited high sensitivity, wide dynamic range, and good reproducibility for all target miRNAs. Overall, these results demonstrate that the integration of a paddle screw-based exosome isolation module with an extension-based nucleic acid detection strategy provides a scalable and biosensor-compatible analytical framework for profiling TNBC-associated exosomal miRNAs, with potential applications in microfluidic liquid biopsy platforms and exosome-based cancer diagnostics.

## 1. Introduction

Exosomes are small extracellular vesicles with diameters ranging from approximately 30 to 150 nm that are secreted by most cell types and play essential roles in intercellular communication, immune regulation, and cellular homeostasis. Numerous studies have shown that cancer cells release exosomes at significantly higher levels than normal cells, making exosomes a rich source of disease-associated molecular information and attractive targets for cancer diagnostics and monitoring [1,2]. Accordingly, considerable efforts have been devoted to the development of reliable exosome isolation technologies, including size-exclusion chromatography, ultracentrifugation, density gradient centrifugation, and immunoaffinity-based capture methods [3,4,5,6,7,8]. Although ultracentrifugation remains the most widely used and is often regarded as the gold standard for exosome isolation, this approach requires large sample volumes, lengthy processing times, and expensive high-speed centrifugation equipment. These limitations hinder its practical application in routine analysis and clinical settings, underscoring the need for alternative exosome isolation strategies that offer higher efficiency, sensitivity, and throughput. In recent years, microfluidic-based exosome isolation platforms and miniaturized bioanalytical systems have attracted growing attention due to their ability to handle small sample volumes, shorten processing times, and enable on-chip integration of isolation and detection steps [9,10,11]. Such microfluidic approaches are particularly attractive for developing compact and automated liquid biopsy systems and for interfacing with biosensor platforms for downstream molecular analysis [12,13,14]. However, many microfluidic exosome isolation devices still suffer from limited capture efficiency due to diffusion-limited mass transfer between exosomes and functionalized surfaces, highlighting the importance of active mixing or flow-enhanced transport strategies to improve isolation performance [15,16,17].

Immunoaffinity-based exosome capture has emerged as a particularly promising approach, as it enables selective isolation of exosomes through specific interactions between surface antigens and corresponding antibodies [18]. This strategy allows enrichment of well-defined exosome subpopulations according to their surface marker expression and facilitates downstream molecular analysis with improved specificity. Exosomes encapsulate a diverse array of biomolecules, including mRNAs, microRNAs (miRNAs), DNA fragments, lipids, and proteins, which collectively reflect the molecular and physiological state of their parent cells [3]. Among these cargos, miRNAs have received increasing attention due to their regulatory roles in gene expression and their strong association with disease progression. Notably, immunoaffinity-based capture schemes are readily compatible with microfluidic channel architectures and surface-functionalized biosensor interfaces, providing a foundation for integrated exosome isolation–detection platforms [10,11].

Among the diseases in which exosome-mediated molecular signaling plays a critical role, triple-negative breast cancer (TNBC) has attracted particular attention due to its aggressive clinical behavior and the lack of well-defined molecular biomarkers. TNBC is an aggressive and clinically challenging subtype of breast cancer characterized by the absence of estrogen receptor, progesterone receptor, and human epidermal growth factor receptor 2 (HER2) expression. Due to the lack of these therapeutic targets, TNBC patients do not benefit from endocrine or HER2-targeted therapies and frequently experience poor clinical outcomes, including early recurrence and a high propensity for distant metastasis [19,20]. Accumulating evidence indicates that TNBC is associated with profound dysregulation of miRNA expression, which plays a pivotal role in driving tumor aggressiveness and therapeutic resistance. In particular, miR-21, miR-106b, and miR-124 have been consistently reported to be up- or down-regulated in TNBC and implicated in key oncogenic processes such as uncontrolled cell proliferation, immune evasion, metabolic reprogramming, and metastatic dissemination. Exosomes secreted by TNBC cells carry these disease-associated miRNAs and faithfully reflect the molecular status of the parent tumor, making TNBC-derived exosomal miRNAs highly informative biomarkers for cancer detection and monitoring. These features further motivate the development of integrated exosome isolation and miRNA sensing platforms for TNBC-oriented liquid biopsy applications [21].

MicroRNAs are short (21–25 nt), endogenous, non-coding RNA molecules that regulate gene expression by binding to complementary sequences in the 3′-untranslated regions of target mRNAs [22]. Dysregulated miRNA expression has been closely linked to cancer initiation, progression, metastasis, immune dysfunction, and therapeutic resistance. Importantly, miRNAs encapsulated within exosomes are shielded from enzymatic degradation, allowing them to remain highly stable in circulating bodily fluids such as blood and urine. This exceptional stability makes exosomal miRNAs particularly attractive candidates for non-invasive liquid biopsy-based diagnostics [23,24,25,26,27]. Tumor-derived exosomes contain distinct miRNA expression patterns that are rarely observed in healthy individuals, further emphasizing their diagnostic and prognostic potential. From a biosensing perspective, the stability and accessibility of exosomal miRNAs make them well-suited targets for integration with nucleic acid-based biosensors and microfluidic molecular diagnostic devices [28,29,30]. A wide range of analytical methods has been developed for miRNA detection, including Northern blotting, microarrays, RT-qPCR, amplification-based strategies, and nanotechnology-enabled approaches [31,32,33,34,35,36,37,38,39,40,41,42]. Among these, RT-qPCR remains the most commonly used technique due to its high sensitivity, quantitative accuracy, and practical applicability [40,41]. However, the short length of miRNAs poses inherent challenges for conventional PCR-based detection, often leading to nonspecific amplification and limited multiplexing capability. To address these issues, various approaches such as stem-loop primers, TaqMan probes, and locked nucleic acid (LNA)-modified oligonucleotides have been introduced [43,44,45,46,47,48]. While these methods provide excellent performance, they often involve high experimental cost, complex assay design, and limited scalability. These limitations present barriers to the direct integration of miRNA detection schemes into compact biosensor platforms and lab-on-a-chip devices, where assay simplicity and robustness are critical [49,50].

In this work, we present an advanced miRNA detection strategy designed to enhance specificity and analytical efficiency through the incorporation of a tailored extension sequence [51]. The proposed assay addresses key limitations of conventional miRNA detection methods that rely on target-specific reverse transcription primers by employing a universal reverse transcription primer for cDNA synthesis, followed by quantitative amplification using universal forward and reverse primers. This unified primer design simplifies assay workflows, reduces primer dependency, and minimizes variability arising from parallel cDNA synthesis for multiple targets. Furthermore, the use of a defined-length amplification template improves reaction consistency and quantitative reliability. Importantly, the paddle screw-based immunoaffinity exosome isolation system employed in this study provides a mechanically driven mixing mechanism that enhances mass transfer between exosomes and antibody-functionalized capture surfaces. This active transport concept is closely aligned with microfluidic mixing and flow-enhancement principles and offers a structurally simple module that can be adapted to microfluidic channels or integrated upstream of biosensor interfaces for automated exosome isolation and molecular analysis. Using this platform, we aim to sensitively and quantitatively detect TNBC-associated exosomal miRNAs—miR-21, miR-106b, and miR-124—isolated from MDA-MB-231 cells, which are strongly implicated in TNBC progression and tumor aggressiveness. By integrating extension-based specificity with a mechanically enhanced exosome isolation strategy, this approach provides a scalable framework for microfluidic-compatible, biosensor-oriented exosomal miRNA analysis, highlighting its potential utility in diagnostic and translational applications.

## 2. Materials and Methods

### 2.1. Reagents and Apparatus

The human triple-negative breast cancer cell line MDA-MB-231 (ATCC^®^ HTB-26™) used for exosome collection was obtained from the American Type Culture Collection (ATCC, Rockville, MD, USA). Exosomes were isolated using the Total Exosome Isolation (TEI) reagent (Invitrogen, Carlsbad, CA, USA), and total RNA, including small RNAs, was extracted using the RNeasy Mini Kit (Qiagen, Valencia, CA, USA) according to the manufacturers’ protocols. The miScript II RT Kit (Qiagen, Hilden, Germany) was used for reverse transcription of polyadenylated miRNAs according to the manufacturer’s protocol. The kit consists of 10× miScript Nucleics Mix, 5× miScript HiSpec Buffer, and a miScript Reverse Transcriptase Mix containing both polyadenylate polymerase (PAP) and reverse transcriptase. The miScript Nucleics Mix provides dNTPs, rATP, and oligo(dT) primers required for cDNA synthesis. Reverse transcription enzyme and PAP were purchased from Invitrogen (Carlsbad, CA, USA). An HB miR Multi Assay Kit™ for RT-qPCR-based miRNA detection was obtained from HeimBiotek, Inc. (Pangyo, Republic of Korea). For PCR product purification, gel extraction, and plasmid isolation, QIAquick^®^ PCR Purification Kit, QIAquick^®^ Gel Extraction Kit, and QIAprep^®^ Spin Miniprep Kit (Qiagen, Hilden, Germany) were used, respectively. HPLC-purified synthetic miRNAs (miR-21, miR-106b, and miR-124), along with their corresponding extension sequences and universal primers for reverse transcription and quantitative PCR, were purchased from Bioneer (Daejeon, Republic of Korea). The nucleotide sequences of all oligonucleotides used in this study are listed in Table 1. Phosphate-buffered saline (PBS, pH 7.4) was purchased from Thermo Fisher Scientific (Waltham, MA, USA). All chemicals and reagents were of analytical grade and used as received without further purification. All aqueous solutions were prepared using ultrapure water obtained from a Milli-Q water purification system (Millipore Corporation, Billerica, MA, USA).

### 2.2. Preparation of Antibody-Conjugated Paddle Screw Devices for Exosome Isolation

**Figure 1:**
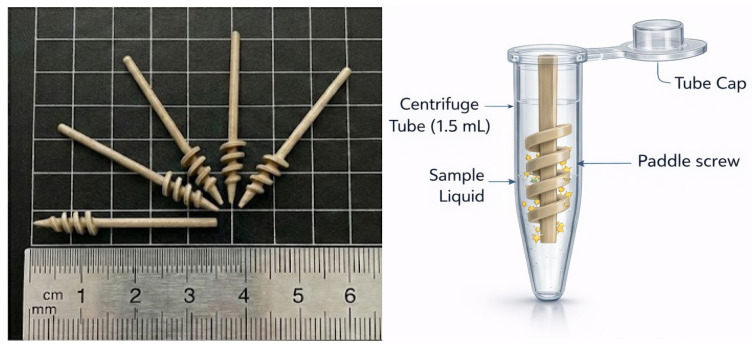
The paddle screw design was selected based on its compatibility with 3D-printing fabrication and its proper fit within a 1.5 mL microtube.

Paddle screw-type devices were fabricated from acrylonitrile butadiene styrene (ABS) using an iSLA550 3D printer (ZRapid Technologies Co., Ltd., Suzhou, China). Among various paddle geometries evaluated, the design shown in Figure 1 was selected due to its compatibility with standard 1.5 mL microcentrifuge tubes and its suitability for reproducible 3D printing. The fabricated paddle screws were sequentially cleaned by sonication in ethanol and double-distilled water (three cycles, 5 min each) at room temperature, followed by drying under a nitrogen stream and storage in a desiccator until further use. Surface activation was performed using a UV–ozone cleaner (144AX-220; Jelight Company, Inc., Irvine, CA, USA) for 10 min. The activated paddle screws were then immersed in a freshly prepared 3% (*v*/*v*) solution of (3-glycidyloxypropyl)triethoxysilane (3-GPTES) in ethanol for 1 h to introduce epoxy functional groups. After silanization, the paddle screws were rinsed with ethanol, dried under nitrogen, and thermally cured at 110 °C for 1 h to stabilize the silane layer. For antibody immobilization, the 3-GPTES-modified paddle screws were incubated in a mixed antibody solution containing anti-CD9 and anti-CD81 (each at 100 μg/mL in phosphate-buffered saline (PBS), pH 7.4) at 4 °C overnight. Following incubation, unbound and physically adsorbed antibodies were removed by rinsing and active rotation of the paddle screws at 500 rpm in PBS and deionized water. To minimize nonspecific protein adsorption during subsequent exosome capture, remaining unreacted surface sites were blocked with 3% bovine serum albumin (BSA) in PBS (pH 7.4).

### 2.3. Immunoaffinity-Based Isolation and Characterization of Cancer Cell-Derived Exosomes

**Figure 2:**
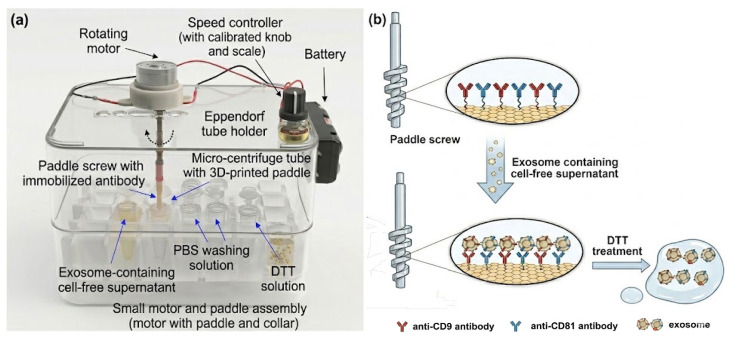
(**a**) Custom-made paddle screw rotating system used for exosome isolation. (**b**) Schematic representation of the working mechanism underlying exosome capture and release using paddle screw devices.

Exosomes were isolated from the human triple-negative breast cancer cell line MDA-MB-231 using two immunoaffinity-based capture approaches targeting the exosomal surface markers CD9 and CD81. For Dyna Beads^®^-based isolation, magnetic beads conjugated with anti-CD9 and anti-CD81 antibodies (Invitrogen, Carlsbad, CA, USA) were used according to the manufacturer’s protocol. For paddle screw-based immunoaffinity isolation, MDA-MB-231 cells were cultured for 24 h, and the conditioned media were collected and sequentially centrifuged at 2000× *g* for 10 min and 15,000× *g* for 15 min to remove cells and cellular debris. The clarified supernatant was further filtered through a 0.22 μm syringe filter (Millipore, Billerica, MA, USA) and transferred to a 1.5 mL microtube. Antibody-conjugated paddle screws were then immersed in the cell-free supernatant and rotated at 200 rpm for 30 min to capture exosomes. After incubation, the paddle screws were transferred to fresh tubes containing phosphate-buffered saline (PBS) and washed by rotation at 500 rpm for 5 min to remove nonspecifically bound components. Captured exosomes were released by transferring the paddle screws into a 50 mM dithiothreitol (DTT) solution and rotating at 200 rpm for 15 min to cleave disulfide bonds between the antibodies and the paddle screw surface. All paddle screw-based isolation steps were conducted using a custom-built rotational system (Figure 2). The exosome-containing solution was subsequently centrifuged at 10,000× *g* for 1 h at 4 °C, and the resulting pellet was resuspended in 1.0 mL of 1× PBS for downstream analyses. Nanoparticle tracking analysis (NTA) was performed using a NanoSight NS300 system (Malvern Panalytical, Malvern, UK) under conditions recommended by the manufacturer, and data acquisition and analysis were carried out with NanoSight NTA software (version 3.3). The total protein concentration of isolated exosomes was quantified using a bicinchoninic acid (BCA) assay kit (Thermo Fisher Scientific, Waltham, MA, USA). Western blot analysis was conducted to confirm the presence of exosomal markers CD9 and CD81.

### 2.4. Poly(A) Tailing-Based miRNA Detection Assay

The quantitative analysis of miRNA expression was conducted using a poly(A) tailing-based RT-qPCR method with the miScript^®^ II RT Kit (Qiagen, Hilden, Germany). A total of 1.0 μg of total human brain RNA was subjected to polyadenylation and reverse transcription in a final reaction volume of 20 μL at 37 °C for 60 min. Enzymatic activity was terminated by heating the reaction mixture at 95 °C for 5 min. Quantitative PCR was carried out using the miScript^®^ SYBR Green PCR Kit (Qiagen, Hilden, Germany). Each 20 μL qPCR reaction contained 2× QuantiTect^®^ SYBR Green PCR Master Mix (including HotStartTaq^®^ DNA polymerase), 10× miScript Universal Primer, 10× miScript Primer Assay, cDNA template, and RNase-free water. The amplification protocol consisted of an initial activation step at 95 °C for 15 min, followed by 40 cycles of 94 °C for 15 s, 55 °C for 30 s, and 70 °C for 30 s. Reverse transcription and real-time PCR were performed using a T100™ Thermal Cycler and a CFX96™ Real-Time PCR System (Bio-Rad Laboratories, Hercules, CA, USA), respectively.

### 2.5. Specific Extension Sequence-Based RT-qPCR Assay for miRNA Detection

A miRNA detection assay based on a specific extension sequence was conducted using a two-step protocol. In the first step, polyadenylation and reverse transcription were carried out simultaneously in a 20 μL reaction containing RT buffer, nucleic mix I (comprising dNTPs, ATP, and RT primer), enzyme mix (0.5 U PAP and 0.5 U reverse transcriptase), and RNase-free water. The reaction was incubated at 37 °C for 10 min and subsequently heated to 95 °C for 5 min to inactivate the enzymes. In the second step, real-time PCR was performed using SYBR Green fluorescence to quantify miRNA levels. Each 20 μL PCR mixture contained cDNA obtained from the first step, nucleic mix II (including the specific extension sequence and primer set), and 2× SYBR Green Master Mix. The PCR cycling conditions consisted of an initial activation at 95 °C for 15 min, followed by 40 cycles of 95 °C for 10 s and 60 °C for 40 s. All reverse transcription and qPCR reactions were carried out using a T100™ Thermal Cycler and a Bio-Rad CFX96™ Real-Time PCR System.

### 2.6. Detection of Exosomal miRNAs Derived from Human Triple-Negative Breast Cancer Cells

Exosomes were collected from the human triple-negative breast cancer cell line MDA-MB-231 using the Total Exosome Isolation (TEI) reagent (Invitrogen, Carlsbad, CA, USA) according to the manufacturer’s protocol. Total RNA enriched with small RNAs was subsequently isolated from the purified exosomes using the RNeasy Mini Kit (Qiagen). The resulting exosomal miRNA samples were serially diluted over a concentration range of 1.0 pg/mL to 100 μg/mL. Exosomal miRNAs were analyzed using both a poly(A) tailing-based miRNA detection assay and a specific extension sequence-based RT-qPCR assay, following the procedures described above, with exosome-derived miRNAs used in place of synthetic miRNA standards. All experiments were performed in triplicate to ensure analytical reproducibility.

## 3. Results and Discussion

### 3.1. Exosome Isolation from MDA-MB-231 Cells and Functional Relevance of TNBC-Associated miRNAs

Exosomes were isolated from cell-free conditioned media of human triple-negative breast cancer (TNBC) MDA-MB-231 cells (ATCC^®^ HTB-26™) using two immunoaffinity-based approaches: commercially available magnetic beads conjugated with anti-CD9 or anti-CD81 antibodies (Dynabeads^®^-CD9 and Dynabeads^®^-CD81), and custom-fabricated paddle screw (PS) devices functionalized with anti-CD9 (PS-CD9), anti-CD81 (PS-CD81), or a combination of both antibodies (PS-DUAL). Prior to immunocapture, conditioned media were subjected to standard pre-clearing steps to remove cells and large debris. The MDA-MB-231 cell line is widely employed as a representative in vitro model of aggressive TNBC [52,53], exhibiting dysregulated expression of multiple microRNAs (miRNAs) associated with tumor progression and metastasis. Among these, miR-21, miR-106b, and miR-124 were selected as representative TNBC-associated miRNAs based on their reported involvement in oncogenic signaling, cell cycle regulation, and tumor suppression, respectively [54,55,56].

**Figure 3:**
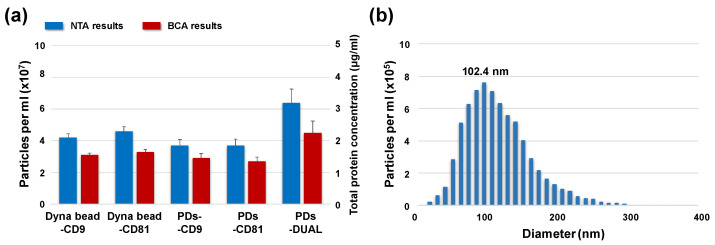
Characterization of exosomes derived from MDA-MB-231 cells. (**a**) Nanoparticle tracking analysis (NTA) and bicinchoninic acid (BCA) protein quantification of isolated exosomes, (**b**) size distribution profiles of exosomes captured using the PDs-DUAL system.

The isolation performance of each platform was quantitatively evaluated using nanoparticle tracking analysis (NTA) and bicinchoninic acid (BCA) protein assays (Figure 3a). The PS-DUAL configuration, employing simultaneous targeting of CD9 and CD81, yielded the highest exosome recovery among the paddle screw-based methods, reaching particle concentrations of approximately 6.4 × 10^7^ particles/mL, which were comparable to those obtained using the optimized commercial Dynabeads^®^ platform. In contrast, PS-CD9 and PS-CD81 devices functionalized with a single antibody exhibited relatively lower isolation efficiencies, underscoring the benefit of multi-marker targeting to account for heterogeneous tetraspanin expression on tumor-derived exosomes. This observation is consistent with previous reports highlighting the phenotypic diversity of exosomal surface markers in cancer-derived vesicles [57]. The paddle device was designed with a screw-like geometry to enhance hydrodynamic mixing during rotation. The helical structure generates both axial and radial flow components, promoting efficient transport of exosomes toward the antibody-functionalized surface and accelerating immunoaffinity capture. In addition to improving mixing during the capture step, the screw-like geometry also facilitates effective washing and drying by promoting fluid circulation and displacement within the tube. Notably, the paddle design is optimized for operation in standard 1.5 mL microcentrifuge tubes, allowing efficient mixing and surface interaction within a confined sample volume without requiring specialized microfluidic equipment. Consistent with this design rationale, a key advantage of the paddle screw-based platform is the substantial reduction in processing time. The total isolation procedure was completed within 1 h, without the prolonged incubation steps commonly required for bead-based immunocapture. Computational fluid dynamics (CFD) simulations further demonstrated that active rotation of the paddle screw at 200 rpm enhanced local mixing efficiency by approximately 13-fold compared with diffusion-dominated conditions [58], thereby promoting more frequent antigen–antibody interactions at the solid–liquid interface. The improved isolation efficiency of the paddle screw device can be attributed to enhanced convective mixing generated by rotational motion, which accelerates antigen–antibody interactions and leads to faster capture kinetics compared with diffusion-limited bead-based systems. NTA measurements indicated that exosomes isolated using PS-DUAL exhibited an average hydrodynamic diameter of approximately 102.4 nm (Figure 3b), consistent with the characteristic size distribution of exosomes reported in the literature. Although the present study focused on milliliter-scale samples, the paddle screw concept could be adapted for smaller volumes through device miniaturization or integration with microfluidic systems, enabling efficient exosome capture in compact analytical platforms.

Beyond isolation performance, the biological relevance of the recovered exosomes was examined by profiling TNBC-associated miRNA cargo. miR-21 is a well-established oncomiR that is frequently overexpressed in TNBC and contributes to tumor aggressiveness through the repression of tumor suppressor pathways involved in apoptosis and immune modulation [59]. Elevated levels of exosomal miR-21 have been correlated with increased metastatic potential and unfavorable clinical outcomes [60]. miR-106b, a member of the miR-106b–25 cluster, plays a critical role in cell cycle progression and proliferative signaling in aggressive breast cancer subtypes [61], and its presence in tumor-derived exosomes is indicative of active oncogenic signaling. In contrast, miR-124 is generally regarded as a tumor-suppressive miRNA that is frequently downregulated in TNBC, contributing to enhanced invasiveness, epithelial–mesenchymal transition, and metastatic dissemination [62]. Accordingly, reduced levels of exosomal miR-124 reflect the loss of tumor-suppressive regulatory mechanisms.

Overall, these results demonstrate that the dual-antibody paddle screw platform enables rapid and efficient isolation of TNBC-derived exosomes while preserving biologically meaningful miRNA cargo. The combined analysis of miR-21, miR-106b, and miR-124 provides a multidimensional molecular readout of TNBC pathobiology, encompassing oncogenic activation, proliferative signaling, and attenuation of tumor-suppressive pathways. This integrated microfluidic–immunoaffinity strategy therefore represents a technically robust and biologically informative approach for exosome-based miRNA profiling, with potential utility in cancer diagnostics and precision medicine applications.

### 3.2. Principle of miRNA Detection Using Specific Bidirectional Extension Sequences

**Figure 4:**
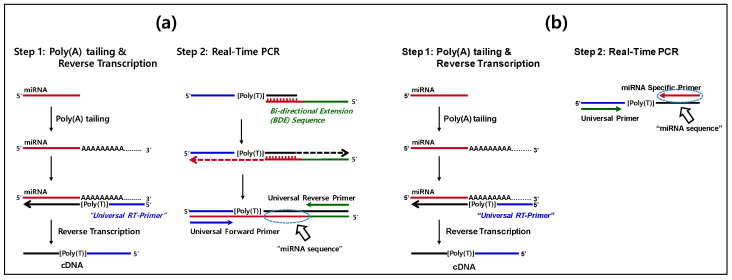
Schematic illustration of RT-qPCR assays for miRNA detection using (**a**) a poly(A) tailing and specific bidirectional extension sequence-based assay and (**b**) a commercial poly(A) tailing assay. Dashed ellipses denote the location of miRNA sequences.

Figure 4a schematically illustrates the miRNA detection strategy employed in this study, which is based on specifically designed extension sequences and represents a modified poly(A)-adaptor RT-qPCR approach optimized for miRNA quantification. Similar to previously reported approaches, the method consists of two sequential reaction steps: reverse transcription and real-time PCR amplification. In the undergop, total RNA containing miRNAs undergoes poly(A) tailing using PAP and ATP as the substrate. The polyadenylated miRNAs are subsequently converted into cDNA through reverse transcription primed by a poly(T)-based adaptor sequence. In this study, a short universal reverse transcription primer (29–34 nucleotides) was used to improve reverse transcription efficiency. Following cDNA synthesis, the resulting cDNA hybridizes with the 3′ end of a specifically designed extension sequence. This hybridization enables dual extension and produces a defined double-stranded PCR template composed of the miRNA-derived cDNA and the extension sequence. In contrast to the commercial poly(A) tailing assay (Figure 4b), which directly uses the single-stranded cDNA product as the PCR template, the extension-based approach generates a newly extended double-stranded template that improves amplification specificity. Subsequent SYBR Green-based RT-qPCR amplification is performed using universal forward and reverse primers. Notably, the forward primer sequence originates from the adaptor used during reverse transcription and partially overlaps with the 5′ region of the extension sequence.

These fundamental differences between the conventional poly(A) tailing assay (Figure 4b) and the extension-based strategy described here contribute to the superior specificity and amplification efficiency of the proposed method. Subsequent SYBR Green-based qPCR is performed using universal forward and reverse primers, as illustrated in Figure 4a. In this configuration, the universal forward primer sequence is embedded within the poly(T) adaptor used during reverse transcription and partially overlaps with the 5′ region of the specific extension sequence. Owing to its simplified primer design and enhanced specificity, this extension-based miRNA detection strategy is broadly applicable to qPCR-based analysis of diverse targets and offers advantages in terms of convenience and cost-effectiveness. Moreover, in contrast to poly(A) tailing assays, which rely on single-stranded cDNA templates, the extension-based approach enables multiplex miRNA detection. This capability arises from the use of double-stranded PCR templates generated through miRNA-specific extension sequences, allowing distinct extension sequences to be independently designed for individual miRNA targets.

In the reverse-transcription step of the extension-based miRNA assay, we employed significantly shorter poly(T)-specific adaptors (29–34 nucleotides) than those used in conventional commercial poly(A) tailing assays (70–80 nucleotides), enabling rapid reverse-transcription kinetics at 37 °C. Under these conditions, cDNA synthesis from poly(A)-tailed miRNAs was completed within 10 min, whereas the conventional poly(A) tailing approach typically requires 60 min.

**Figure 5:**
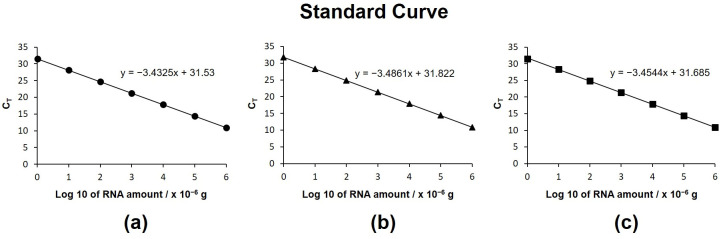
Influence of reverse-transcription time on assay performance for miR-21. C_T_ values plotted against log10-transformed target miRNA concentrations for reaction times of (**a**) 10 min, (**b**) 30 min, and (**c**) 60 min, with linear extrapolation. Each data point represents a single measurement, as the obtained signals showed highly consistent trends across the measurement range.

Figure 5 presents standard curves obtained from RT-qPCR amplification of serial dilutions of synthetic miR-21 (UAGCUUAUCAGACUGAUGUUGA) using reverse-transcription reaction times of 10, 30, and 60 min. For all reaction durations, a linear relationship was observed between the threshold cycle (C_T_) values and the logarithm of total RNA concentration, and no statistically significant differences were detected among the three curves. These results indicate that sufficient cDNA synthesis was achieved within 10 min using the short, target-specific poly(T) adaptor. By contrast, in the commercial poly(A) tailing assay, shortening the reverse-transcription time from 60 min to 10 min resulted in a noticeable increase in C_T_ values, reflecting reduced reverse-transcription efficiency. The enhanced reaction rate observed in the extension-based assay can be attributed to the rapid diffusion and improved hybridization efficiency of the shorter poly(T)-specific adaptors. Although cDNA generated using short poly(T) adaptors consists of relatively short sequences (approximately 50–60 nucleotides, considering the miRNA length of 20–25 nucleotides), which are generally insufficient to serve as stable PCR templates, the extension-based miRNA assay overcomes this limitation. Specifically, the short cDNA is dually extended by a uniquely designed specific extension sequence (60–90 nucleotides), resulting in the formation of a long, double-stranded PCR template suitable for robust and reliable amplification.

### 3.3. Analysis of Exosomal miRNAs from MDA-MB-231 Cells Using an Extension-Based miRNA Assay

**Figure 6:**
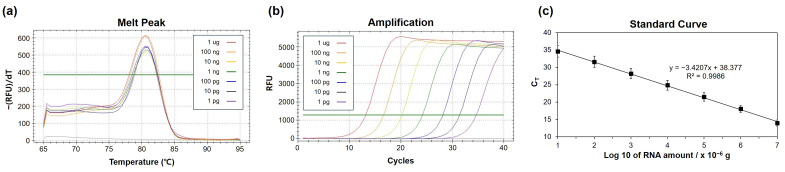
RT-qPCR characterization of miR-106b in total human brain RNA. (**a**) Melting curves (65–95 °C). (**b**) Amplification curves across a seven-log dilution series of miR-106b. (**c**) Standard curve of Ct versus log10 miRNA amount (R^2^ = 0.9986; slope = −3.421; efficiency = 96.0%). Each concentration was measured in triplicate.

Among the three TNBC-associated miRNAs investigated in this study (miR-21, miR-106b, and miR-124), miR-106b (UAAAGUGCUGACAGUGCAGAU) was selected as a representative target to evaluate the dynamic range and analytical sensitivity of the proposed extension-based miRNA quantification strategy. Total RNA extracted from exosomes derived from the MDA-MB-231 human triple-negative breast cancer cell line was used as the biological matrix. Quantitative detection of miR-106b was achieved over a concentration span of seven orders of magnitude, from 1.0 pg to 1.0 µg of total RNA, based on real-time monitoring of SYBR Green fluorescence. As shown in Figure 6a, a single, well-defined dissociation peak was observed at approximately 80.5 °C, indicating highly specific amplification of the miR-106b target. The corresponding amplification profiles across this wide concentration range are presented in Figure 6b. Specificity of the qPCR products was verified by melting curve analysis. The extension-based SYBR Green RT-qPCR assay further demonstrated strong quantitative performance, exhibiting a robust linear relationship between the logarithm of the input RNA amount and the corresponding C_T_ values. The standard curve generated from serial dilutions of total RNA (Figure 6c) reliable amplification over the range of 10^2^–10^6^ pg, with a calculated PCR efficiency of 96% based on a slope of −3.42. The correlation coefficient (R^2^ = 0.9986) confirmed excellent linearity and quantitative accuracy. These results indicate that the assay provides a dynamic range of at least seven orders of magnitude and is capable of detecting miR-106b at levels as low as 0.1 pg of total RNA in the PCR reaction.

**Figure 7:**
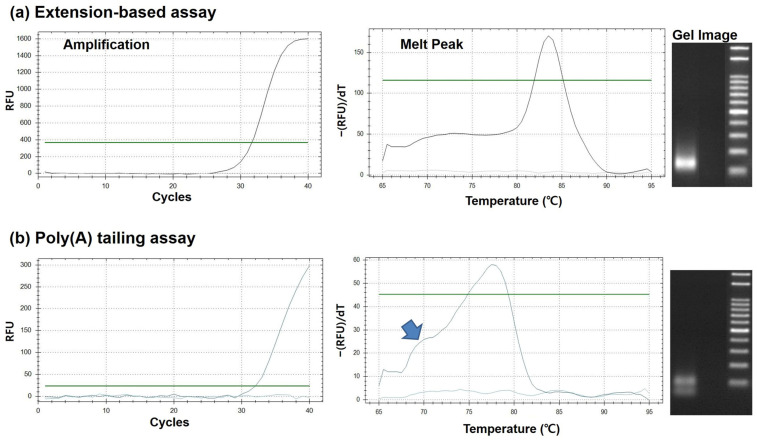
Comparison of assay specificity for miR-124 derived from exosomes of MDA-MB-231 human triple-negative breast cancer cells: (**a**) extension sequence-based RT-qPCR assay and (**b**) commercial poly(A) tailing-based assay. Amplification profiles, melting curve analyses, and agarose gel electrophoresis results are shown.

The analytical performance of the proposed extension-based miRNA assay was evaluated by comparing its specificity with that of a commercially available poly(A)-tailing method (AM1350, Thermo Fisher Scientific, Waltham, MA, USA). This comparison was conducted using three representative miRNAs isolated from the human triple-negative breast cancer cell line MDA-MB-231. The comparative analytical results for miR-124 (UAAGGCACGCGGUGAAUGCC) are summarized in Figure 7, while the corresponding data for miR-21 and miR-106b are provided in the Supplementary Information (Figure S1). Real-time PCR amplification curves demonstrated that the threshold cycle (C_T_) values obtained using the extension-based assay were comparable to those obtained with the poly(A)-tailing assay for all three miRNAs, indicating similar amplification efficiencies between the two methods. For example, in the case of miR-124, the C_T_ values were 31.99 for the extension-based assay and 31.66 for the poly(A)-tailing assay. Similar trends were observed for miR-21 and miR-106b, confirming that differences in assay performance were not attributable to variations in PCR efficiency. Despite comparable amplification efficiencies, pronounced differences in amplification specificity were observed. As shown in Figure 7a, melting curve analysis revealed that the extension-based assay consistently produced a single, sharp melting peak for all three miRNAs, indicative of highly specific target amplification. In contrast, the poly(A)-tailing assay (Figure 7b) exhibited additional shoulder peaks adjacent to the main melting peak, particularly evident for miR-124, suggesting the presence of nonspecific amplification products. The superior specificity of the extension-based assay was further corroborated by agarose gel electrophoresis. While amplification products generated using the poly(A)-tailing method showed weak, smeared, or nonspecific bands—likely arising from polymerase slippage or nonspecific primer annealing associated with long poly(T) sequences [63]—the extension-based assay produced distinct and well-resolved bands at the expected amplicon sizes for miR-21, miR-106b, and miR-124. Taken together, these results demonstrate that the extension-based miRNA assay offers markedly improved specificity over the conventional poly(A)-tailing method while maintaining comparable amplification efficiency. The consistent performance observed across miR-21, miR-106b, and miR-124 underscores the robustness and general applicability of the proposed assay for sensitive and specific miRNA detection.

## 4. Conclusions

This study demonstrates an integrated analytical strategy that combines paddle screw-based immunoaffinity exosome isolation with an extension-based RT-qPCR assay for exosomal miRNA profiling. This integrated approach addresses key limitations of conventional exosome isolation and miRNA detection methods, particularly the lengthy processing times and diffusion-limited capture efficiency that often restrict their practical implementation in rapid bioanalytical systems. The antibody-functionalized paddle screw enabled rapid and efficient isolation of exosomes from the MDA-MB-231 human triple-negative breast cancer cell line, achieving isolation performance comparable to commercial magnetic bead-based methods while substantially reducing processing time. The isolated vesicles were verified as exosomes by nanoparticle tracking analysis and immunoblotting of canonical surface markers. Importantly, the mechanically driven paddle-based isolation concept provides a simple and scalable framework that is readily adaptable to microfluidic devices and surface-based biosensor architectures. The screw-like paddle geometry enhances hydrodynamic mixing and promotes efficient antigen–antibody interactions, enabling rapid exosome capture within a compact sample volume.

Following exosome isolation, three TNBC-associated exosomal miRNAs—miR-21, miR-106b, and miR-124—were quantitatively analyzed using a specific extension sequence-based RT-qPCR assay, which exhibited high sensitivity, broad dynamic range, and enhanced specificity compared with conventional poly(A)-tailing methods. By minimizing nonspecific amplification and improving template fidelity, the proposed detection strategy is well suited for integration into compact nucleic acid biosensing platforms. The improved specificity of the present amplification strategy may be particularly advantageous for multiplex miRNA profiling and liquid biopsy applications, where closely related miRNA sequences coexist and nonspecific amplification can compromise analytical accuracy.

Overall, the presented workflow offers a rapid, sensitive, and cost-effective approach for exosomal miRNA analysis and provides a practical foundation for the development of integrated microfluidic biosensor systems for liquid biopsy-based cancer diagnostics and precision oncology. In particular, the device architecture and assay format offer opportunities for future integration with miniaturized bioanalytical systems, including microfluidic platforms and biosensor-based detection technologies for exosome analysis.

## Figures and Tables

**Table 1:** Oligonucleotide sequence used in this experiment.

No	miRNAs		Sequences (5′ → 3′)
1	miR-21	miRNA	UAG CUU AUC AGA CUG AUG UUG A
		Extension	GCA GAC GGC TCA GGA TAC GGA TAA CGG CTA
			CTC CGT GTT TGA GCA GTC ACT GCT GCG GTA
			TAT CGT AGC TTA TCA GAC TGA TGT TGA
2	miR-106b	miRNA	UAA AGU GCU GAC AGU GCA GAU
		Extension	GCA GAC GGC TCA GGA TAC GGA TAA CGG CTA
			CTC CGT GTT TGA GCA GTC ACT GCT GCG GTA
			TAT CGT AAA GTG CTG ACA GTG CAG AT
3	miR-124	miRNA	UAA GGC ACG CGG UGA AUG CC
		Extension	GCA GAC GGC TCA GGA TAC GGA TAA CGG CTA
			CTC CGT GTT TGA GCA GTC ACT GCT GCG GTA
			TAT CGT AAG GCA CGC GGT GAA TGC C
4	Universal RT primer		TAG GCG GAA TCG GTA GTA ATT TTT TTT TTT T
5	Universal forward primer		TAG GCG GAA TCG GTA GTA A
6	Universal reverse primer		GCA GAC GGC TCA GGA TAC G

## Data Availability

Data are contained within the article.

## Supplementary Materials

The following supporting information can be downloaded at: https://www.mdpi.com/article/10.3390/mi17030362/s1, Figure S1: Comparison of assay specificity for miR-21 and miR-106b derived from exosomes of MDA-MB-231 human triple-negative breast cancer cells.
