# Supplementary material for: Isolation of Exosomes from MDA-MB-231 Cells Using a Paddle Screw System and Detection of TNBC-Associated Exosomal miRNAs
Source: Micromachines (Basel). 2026 Mar 16;17(3):362. doi: 10.3390/mi17030362 (PMC13028636; doi:10.3390/mi17030362)

Supplementary Information

1) Comparison of assay specificity for miR-21 and miR-106b derived from exosomes of MDA-MB-231 human triple-negative breast cancer cells: Amplification profiles, melting curve analyses, and agarose gel electrophoresis results are shown.

Poly(A) tailing assay | **Extension-based assay**

miR-21

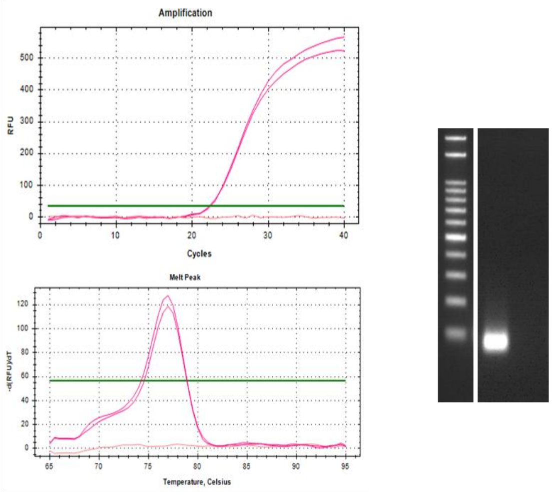

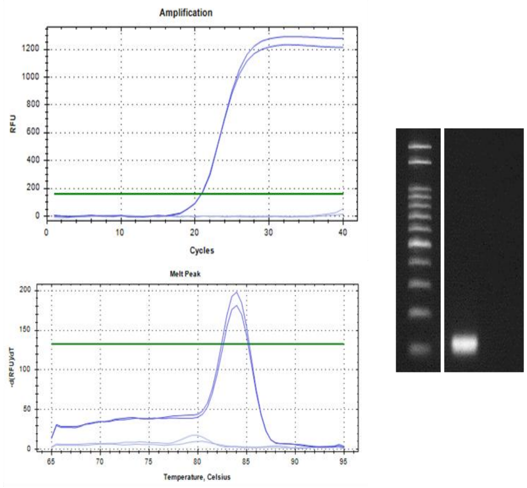

Poly(A) tailing assay | **Extension-based assay**

miR-106b

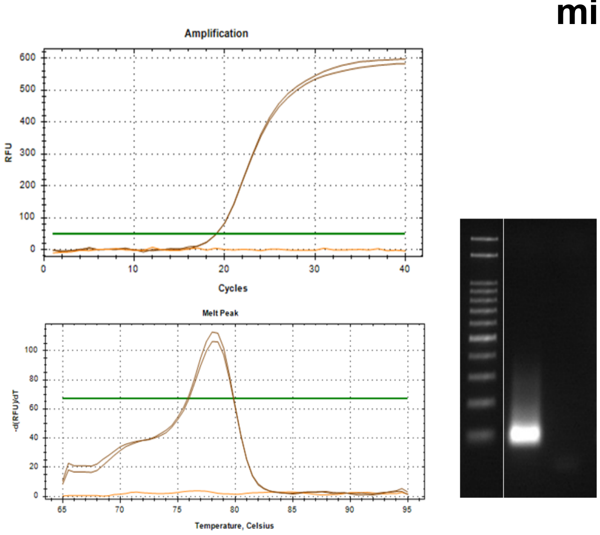

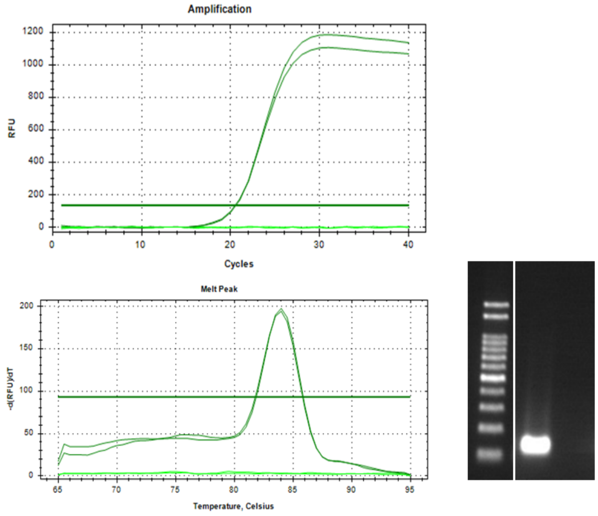

Supplement: Supplementary file 1 [file micromachines-17-00362-s001.zip › micromachines-4169482-supplementary.pdf]
